# Heart rate and insula activity increase in response to music in individuals with high interoceptive sensitivity

**DOI:** 10.1371/journal.pone.0299091

**Published:** 2024-08-22

**Authors:** Toru Maekawa, Takafumi Sasaoka, Toshio Inui, Alan S. R. Fermin, Shigeto Yamawaki

**Affiliations:** 1 Center for Brain, Mind and KANSEI Sciences Research, Hiroshima University, Minami-Ku, Hiroshima, Japan; 2 Kyoto University, Sakyo-ku, Kyoto, Japan; RIKEN CBS: RIKEN Noshinkei Kagaku Kenkyu Center, JAPAN

## Abstract

Interoception plays an important role in emotion processing. However, the neurobiological substrates of the relationship between visceral responses and emotional experiences remain unclear. In the present study, we measured interoceptive sensitivity using the heartbeat discrimination task and investigated the effects of individual differences in interoceptive sensitivity on changes in pulse rate and insula activity in response to subjective emotional intensity. We found a positive correlation between heart rate and valence level when listening to music only in the high interoceptive sensitivity group. The valence level was also positively correlated with music-elicited anterior insula activity. Furthermore, a region of interest analysis of insula subregions revealed significant activity in the left dorsal dysgranular insula for individuals with high interoceptive sensitivity relative to individuals with low interoceptive sensitivity while listening to the high-valence music pieces. Our results suggest that individuals with high interoceptive sensitivity use their physiological responses to assess their emotional level when listening to music. In addition, insula activity may reflect the use of interoceptive signals to estimate emotions.

## Introduction

Interoception, the neural sensation of internal bodily states [1,2], has become a topic of significant interest owing to its crucial role in various aspects of human behavior, including emotion, decision-making, social interaction, and mental health [3–9]. Recent studies have revealed that individuals with high interoceptive sensitivity experience a significant decrease in heart rate when presented with emotional images [10,11]. Additionally, it has been shown that heart rate and other cardiovascular parameters during emotional image observation differ based on the level of interoceptive sensitivity [12].

These findings suggest that the physiological responses of an individual to emotional stimuli are contingent on their level of interoceptive sensitivity, which is defined as their ability to perceive and report physiological signals. However, despite these significant findings, some behavioral studies have not found a clear relationship between interoceptive sensitivity and physiological responses [13,14]. For example, it has been reported that there is no discernible difference in heart rate or skin conductance in response to emotional videos between groups with high and low interoception sensitivity [13].

One possible explanation for these inconclusive results could be the impact of subjective preferences for emotional stimuli on the physiological responses. For instance, studies on physiological responses to music have shown that music genre preferences can influence the strength of physiological responses to music, with weaker physiological responses to non-preferred music [15]. Therefore, we hypothesize that the subjective emotional intensity of stimuli is an important factor to elicit emotion-related physiological responses depending on individual differences in interoceptive sensitivity.

In the cerebral cortex, the insula is a structure implicated in interoceptive information processing, including heartbeat, breathing, hunger, pain, thirst, and stomach sensations [16,17]. The insula is also implicated in higher-order cognitive processes, such as the emergence of interoceptive awareness [3,4,18–22], and in emotional responses to emotion-eliciting stimuli, including auditory signals such as music [23–25]. For example, insula activity has been reported while listening to music stimuli [23]. Anatomically, the insula is subdivided into three neuroanatomical modules based on its cytoarchitectural organization: anterior agranular insula, mid-dorsal dysgranular insula, and posterior granular insula [26,27].

Despite research on the neural basis of interoceptive sensitivity, it has been difficult to consistently identify which, if any, of these insula modules have a specialized role in predicting the impact of individual differences in interoceptive sensitivity on emotional responses. Previous studies have reported correlations between interoceptive sensitivity and activity in the right anterior [16,28] or mid insula [29], as well as decreased network centrality in the right posterior insula in participants with high interoceptive sensitivity [30]. Although there has been considerable progress in understanding the roles of the insula in interoception, these findings have been contradictory, and further research is needed to determine whether activity in distinct insula modules is associated with individual differences in interoceptive sensitivity, as well as emotional and physiological responses.

The present study aimed to elucidate two open questions on the neural substrates involved in the relationship between individual differences in interoceptive sensitivity and emotional responses to music stimuli. Firstly, it aimed to investigate whether individual differences in interoceptive sensitivity influence the relationship between subjective emotional intensity and physiological signals in response to music stimuli. Secondly, the study aimed to explore whether individual differences in interoceptive sensitivity have an impact on insula activity during emotion processing, and if so, which specific insula subregions are involved.

To test these hypotheses, we conducted a functional magnetic resonance imaging (fMRI) experiment to record participants’ brain activity and heart rate while they listened to various music pieces. We used musical stimuli for two reasons. First, it is known that musical stimuli can induce large physiological changes. A study comparing the effects of visual and musical stimuli demonstrated that while the recognition of emotions from visual stimuli was more precise, exposure to musical stimuli resulted in more pronounced changes in electrodermal activity (EDA), heart rate, and brain wave amplitude. [31]. Furthermore, individuals often describe their responses to music using terms related to bodily sensations, such as ’chills’ and ’shivering’ [32,33]. These descriptions indicate that the experience of listening to music is closely tied to changes in physiological states. Second, there is a significant overlap in the neural representation of interoceptive information and the emotional experience induced through music, with the insula being implicated in the integration of physiological responses with emotional experience [23]. Therefore, musical stimuli are suitable for the present study to investigate whether the relationship between subjective emotional intensity and physiological responses is modulated by individual differences in interoceptive sensitivity.

In addition to the fMRI experiment, we also conducted tasks outside of the MRI to measure participant interoceptive sensitivity. The most common psychophysical measure of interoceptive sensitivity is the heartbeat counting task, in which participants silently count the number of heartbeats within a certain timeframe. However, some literature questions the validity of this task [34–36], and some studies have focused on the heartbeat discrimination task [37,38], in which participants listen to a sound stimulus and assess whether it matches the timing of their heartbeat. In the present study, the participants performed both tasks. We then compared individual differences in physiological responses, brain activity during music listening, and interoceptive sensitivity.

## Materials and methods

### Participants

The study involved 52 participants (31 females, 21 males, age range: 18–35 years, average age: 22.6 ± 2.81 years). The participants were recruited between August and December 2019 through Sona-systems (https://sona-systems.com) operated by the Center of KANSEI Innovation at Hiroshima University. All participants were right-handed, were not professional musicians, and had no musical background. Three participants were excluded from the analysis due to a malfunction in the physiological recording.

The participants provided written informed consent in accordance with the Declaration of Helsinki and received a monetary reward for their participation. The study was approved by the research ethics committee of Hiroshima University (approval number: E-965). All information collected for this study will be held in strict confidence and has been anonymized with all participants having been assigned a numeric code. Only the researcher conducting the study had access to such information.

### Heartbeat counting task

In the heartbeat counting task, the participants counted and reported the number of heartbeats between two beep sounds in six trials. Each trial consisted of a variable time interval of 25, 30, 35, 40, 45, or 50 s between beeps. During the task, a 3-lead electrocardiograph (ECG) was attached to the chest of the participants and the ECG was recorded at 1000 Hz using a Biopac MP160 System (Biopac Systems, Goleta, CA). The heartbeat counting task was performed outside the MRI scanner. The participants sat alone in a soundproof room and performed the task—described below—according to display and audio instructions.

From the participants’ responses, the interoceptive accuracy (IA) score was calculated using the following formula:

$$IA=1/6\sum 1-\left|{nbeats}_{reported}-{nbeats}_{recorded}\right|/{nbeats}_{recorded}$$

Here, *nbeat_recorded_* represents the correct number of heartbeats, and *nbeat_reported_* represents the number of heartbeats reported by the participant.

### Heartbeat discrimination task

In the heartbeat discrimination task, participants are asked to determine whether the timing of the beep sound and one’s own heartbeat are synchronized when a beep sound is slightly delayed from the actual heartbeat [39]. A beep sound was presented immediately after the participant’s R-peak was detected with ECG (0 ms condition), or after a 150-, 300-, and 450-ms delay from the R-peak timing. In each trial, the participants listened to 10 beep sounds and answered whether or not the beep sound timing matched their heartbeat. Six trials under each condition were performed, totaling 24 trials.

**Fig 1:**
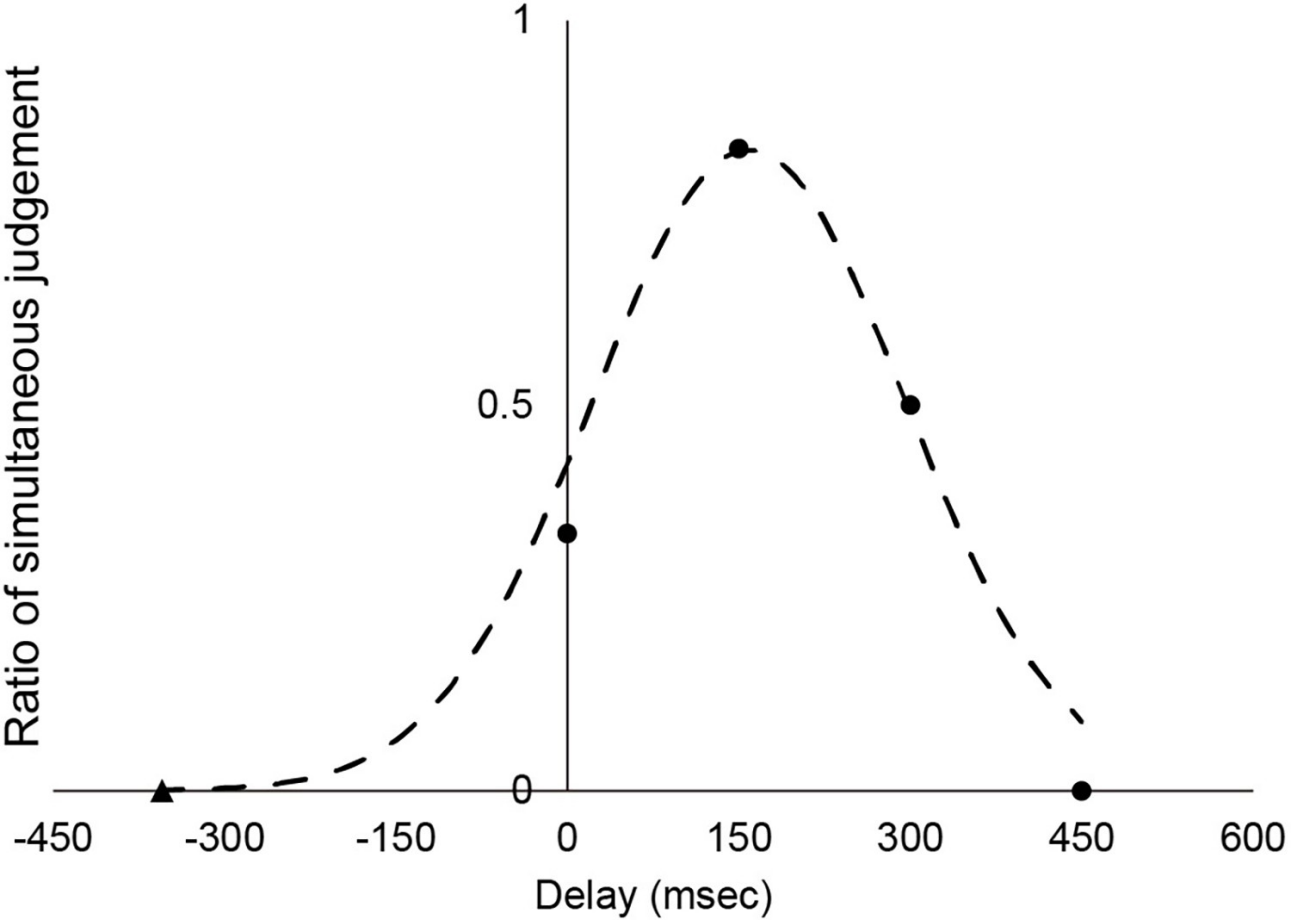
Schematic diagram of the analysis of the heartbeat discrimination task. The horizontal axis shows the delay time of the beep from R-peak, while the vertical axis shows the percentage of trials in which the participants responded that the timing was matched. The triangular symbol indicates the extrapolated condition considering the periodicity of the heartbeat (see details in the text). The dotted line shows the Gaussian approximation.

In the heartbeat discrimination task, the participants do not always feel that their heartbeats are synchronized with the sounds when there is no delay [38,40]. In addition, since the heartbeat is periodic, it might be impossible to distinguish whether the stimulus is delayed or advanced relative to the heartbeat. To address this issue, we created a novel heartbeat discrimination index. To characterize participant performance, we approximated the Gaussian function to the synchronous judgment ratio (Fig 1). In this fitting, the periodicity of the heartbeat was dealt with as follows: In the case of a participant with an R-R interval (RRI) of 800 ms, for instance—because the heartbeat occurs in an almost constant cycle—the 450-ms sound delay is indistinguishable from the 350-ms sound advance. Therefore, the response data under the 450-ms delay can also be treated as relevant data when the sounds are presented 350 ms earlier than the heartbeats. Each participant’s data at five points (450 –RRI ms, 0 ms, 150 ms, 300 ms, and 450 ms) were fitted to the following Gaussian function:

$$Simultaneous\;judgement\;ratio=A\times exp\left(-\frac{{\left(delay-\mu \right)}^{2}}{\sigma^{2}}\right)+b$$

There are four fitting parameters: *A*, *μ*, *σ*, and *b*. If a participant has good heartbeat sensitivity, the Gaussian shape should be higher and sharper. Therefore, we used amplitude *A* and variance *σ* as indicators of interoceptive sensitivity (IS) for subsequent analyses. The MATLAB code used for these calculations is provided in the supporting information.

### Music listening task

Musical stimuli were selected from the music pieces used by Proverbio and Manfrin [41]. We selected 10 tonal music pieces and 10 atonal music pieces (Table 1). The choice to include both tonal and atonal compositions, along with pitch-shifted tonal pieces, was driven by the aim to elicit a broad spectrum of emotional responses and to account for individual variances in musical preferences and familiarity. In addition, for the tonal music pieces, two variations were made by changing the pitches to one half note higher and three half notes lower; then, a music piece was created by synthesizing them [42]. Therefore, a total of 30 music pieces were used, including 10 tonal, 10 atonal, and 10 pitch-shifted tonal music pieces.

**Table 1:** List of music used in the experiment.

Type	Composer	Title
Tonal	Beethoven, Ludwig	Fourth Movement of Symphony No. 5 in C major (op. 67) (last minute of The coda: Allegro)
	Mozart, Wolfgang Amadeus	The Marriage of Figaro–Overture in D major (K492)
	Mendelssohn, Bartholdy Felix	First Movement of Symphony No.4 “Italian” in A major (op.90)
	Mahler, Gustav	Symphony no. 5 in F major (Adagietto)
	Ravel, Maurice	Second Movement of Concert in G major
	Bach, Johann Sebastian	Second Movement of Concert for two Violins in D minor (BWV 1043) (from measure 10)
	Mahler, Gustav	Der Abschied from “The Song of the Earth” in C-minor
	Holst, Gustave	The Planets: Mars, the bringer of war in C minor (op. 32)
	Beethoven, Ludwig van	Piano sonata No. 14 in C-sharp minor, First Movement. (op.27, No.2)
	Beethoven, Ludwig van	Symphony No. 5, First Movement in C minor (op. 67)
Atonal	Cage, John	Fontana Mix, for magnetic tape
	Maderna, Bruno	Serenade for a satellite
	Pärt, Arvo	Cantus in Memoriam of Benjamin Britten (the opening passage, after the first 15 s)
	Ives, Charles	The Unanswered question
	Berg, Alban	Last orchestra interlude from the last scene of Wozzeck
	Ligeti, György	Concert for cello and orchestra
	Donatoni, Franco	Duo Pour Bruno (ninth panel, from 14’ 37”)
	Petrassi, Goffredo	(Chorus of the Dead) Coro di morti
	Boulez, Pierre	Second piano sonata
	Kurtag, Gyorgy	String Quartet No. 1

The participants listened to a music piece while undergoing the fMRI. They wore earplugs and headphones with a noise-canceling function (OptoActive, Optoacoustics Ltd, Or Yehuda, Israel). In addition, a photoplethysmogram (PPG) was recorded from the participants’ left index finger at 1000 Hz using the Biopac MP 150 system. In each trial, after a presentation of a fixation cross for 5 s, the participants listened to a music piece for 30 s.

After listening to each music piece, the participants were asked to assess the strength of their emotional response. The term ’Kando’ in Japanese was used for this purpose, primarily indicating the extent of the positive emotions they felt elicited by the music piece. This assessment was conducted using a visual analog scale ranging from 0 to 1, where higher values indicated a stronger emotional response. Each music piece was presented once, and the participants performed 30 trials. The experiment was divided into two sessions of 15 trials, with a 10-minute break between sessions.

Pulse rate data were processed using a fourth-order Chebyshev Type II filter with a cut-off frequency of 0.5–5 Hz to enhance signal clarity. Peak detection in the filtered PPG data was conducted using MATLAB’s findpeaks function (Mathworks, USA). In calculating the RRIs, data points exceeding ± 3 median absolute deviations were excluded to ensure measurement accuracy and consistency. The detailed script used for this analysis is provided in the supporting information.

### fMRI data acquisition and analysis

A 20-channel head coil 3T MRI system (Siemens MAGNETOM Skyra, Siemens Ltd., Erlangen) was used for data acquisition. Before the experiment, a high-resolution T1-weighted structural scan was obtained using three-dimensional magnetization with a rapid gradient echo imaging sequence (TR = 2300 ms, TE = 2.98 ms, flip angle = 9°, field of view = 256 mm, voxel size 1 × 1 × 1 mm, 176 slices).

The functional images were obtained using an echo-planar T2*-weighted multiband gradient echo sequence (TR = 1000 ms, TE = 30 ms, flip angle = 80 degrees, field of view = 192 mm, voxel size 3.0 × 3.0 x 3.2 mm, 42 slices, acceleration factor 3). For each session, 707–902 functional images were obtained. The first 10 functional images for each session were excluded from the analysis to allow for T1-equilibration. Images were preprocessed with standard routines implemented in the Statistical Parametric Mapping software SPM12 (Wellcome Department of Cognitive Neurology, UK; https://www.fil.ion.ucl.ac.uk/spm). Preprocessing included realignment, normalization to the Montreal Neurological Institute template, and smoothing with a 3D Gaussian filter (full width at half maximum 8 mm).

After preprocessing, the first-level analysis was conducted using a general linear model (GLM) for each participant. The GLM comprised regressors for the periods during the presentation of a music piece, the presentation of fixation before the presentation of the music piece, and the periods during a response. To identify the effect of emotional experience, participant music valence ratings were entered into the model as a parametric modulator. Six head motion parameters were entered as nuisance regressors. We then obtained the parametric modulation contrast images for the subjective ratings of valence level during the presentation of music.

The parametric modulation contrast images for each participant were entered into the group analysis using a random-effect model with a one-sample *t*-test. For the whole-brain analysis, the uncorrected significance level was *p* < 0.001 at the voxel level, and *p* < 0.05 with family-wise error correction at the cluster level.

Next, we conducted a region of interest (ROI) analysis to investigate the effects of individual differences in interoceptive sensitivity. We defined six subregions of the insula for each hemisphere using the Brainnetome Atlas [43]: hypergranular, ventral agranular, dorsal agranular, ventral dysgranular and granular, dorsal granular and dorsal dysgranular insula. For ROI analysis, we used contrast estimates averaged over voxels of each insula subregion in the parametric modulation contrast images for valence levels. The *P*-value threshold was corrected with false discovery rates for multiple comparisons (*p* < 0.05).

## Results

### Heartbeat tasks

The average IA score for the heartbeat counting task was 0.73 ± 0.22. For the heartbeat discrimination task, Gaussian parameters were estimated for each participant, and the average values for each parameter were as follows: *A*: 0.53 ± 0.24, *μ*: 171 ± 139 ms, *σ*: 150 ± 89 ms, and *b*: 0.31 ± 0.19. We then performed Pearson correlations to analyze the relationship between IA and the parameters of the heartbeat discrimination task.

These analyses revealed a positive correlation between IA and the amplitude parameter *A* (*r* = 0.34, *t* (47) = 2.48, *p* = 0.021). No significant relationships were observed between IA and the parameters *μ (r* = -0.07, *t* (47) = 0.45, *p* = 0.65), *σ (r* = 0.07, *t* (47) = 0.45, *p* = 0.65), or *b (r* = -0.11, *t* (47) = 0.79, *p* = 0.43).

### Music valence associated with pulse rate during the music-listening task

First, we investigated whether participants’ music valence ratings were associated with the degree of pulse rate modulation during the presentation of the music piece. The music-modulated pulse rate was calculated as the difference between the average pulse rate during the presentation of the fixation cross and that during the presentation of a music piece. Next, we used a median split method to sub-divide the 30 trials into three levels for each participant based on their music valence ratings: high-valence trials, 10 trials in which the valence score was the highest; middle-valence trials, 10 trials in which the valence score was between low and high; and low-valence trials, 10 trials in which the valence score was the lowest.

A one-way repeated-measures analysis of variance (ANOVA) with a factor of valence level (high-valence, middle-valence, and low-valence trials) was performed on the average music-modulated pulse rate. This analysis revealed a significant main effect of valence level on pulse rate (*F* (1, 48) = 5.67, *p* = 0.021). The post-hoc test with Bonferroni correction revealed a significantly higher pulse rate in the high-valence trials than in the low-valence trials (*p* = 0.049). In contrast, a one-way repeated-measures ANOVA with a factor of music type (tonal, atonal, and discorded) revealed no significant main effect (*F* (1, 48) = 0.13, *p* = 0.73).

### Individual differences in interoceptive sensitivity predict pulse rate

Next, to examine the association of interoceptive sensitivity with music valence level and pulse rate, we divided the participants into two groups according to their amplitude parameters derived from the heartbeat discrimination task, that is, fitting parameter *A*. Since *A* had a distribution close to the normal distribution (Kolmogorov–Smirnov test, *p* = 0.30), we divided the participants into two groups with the same degree of variation by the median split (equality of variance, *F* (23, 24) = 0.84, *p* = 0.69).

**Fig 2:**
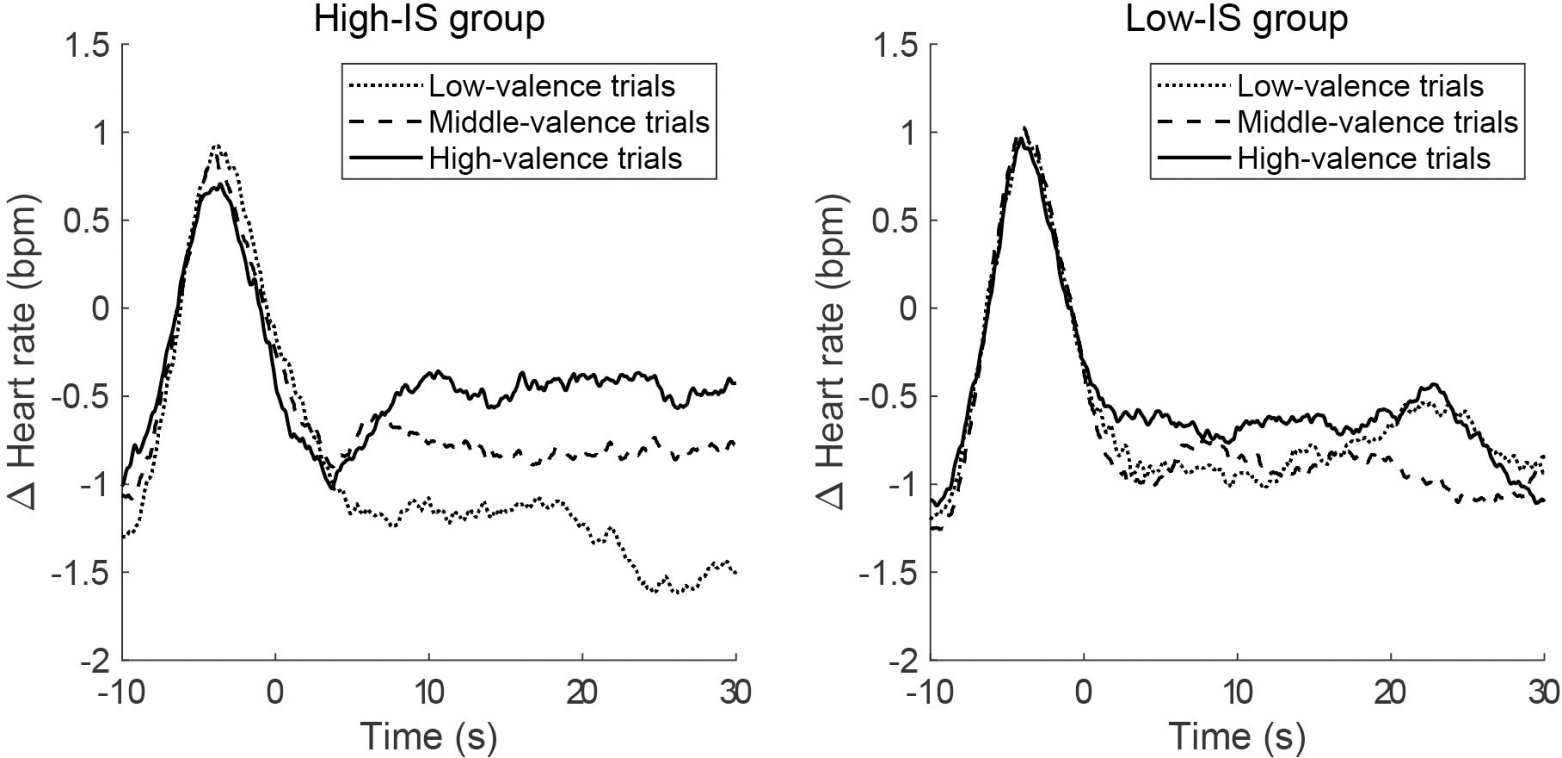
Changes in pulse rate grouped by interoceptive sensitivity defined by heartbeat discrimination task performance. The participants were divided into two groups based on the heartbeat discrimination task amplitude parameter. The graph on the left shows the average result of the high-IS group, while the graph on the right shows the average result of the low-IS group. The vertical axis shows the change in pulse rate based on the fixation time before the trial. The zero on the horizontal axis indicates the time when the music started. IS, interoceptive sensitivity.

Based on the median of *A*, 25 participants with *A* above the median were classified into the high interoceptive sensitivity (high-IS) group, whereas the remaining 24 participants were classified into the low interoceptive sensitivity (low-IS) group. The average pulse rate for each interoceptive sensitivity group is shown in Fig 2.

A two-way repeated measures ANOVA revealed a significant interaction between the interoceptive sensitivity group and the valence level with regard to the average pulse rate (*F* (1, 47) = 8.08, *p* = 0.007) and a significant effect of music valence level (*F* (1, 47) = 6.21, *p* = 0.016). No significant effect was observed in the interoceptive sensitivity group (*F* (1, 47) = 0.03, *p* = 0.57). The post-hoc test with Bonferroni correction revealed a significant difference between the high-valence and the low-valence trials only among the participants in the high-IS group (*p* = 0.012).

**Fig 3:**
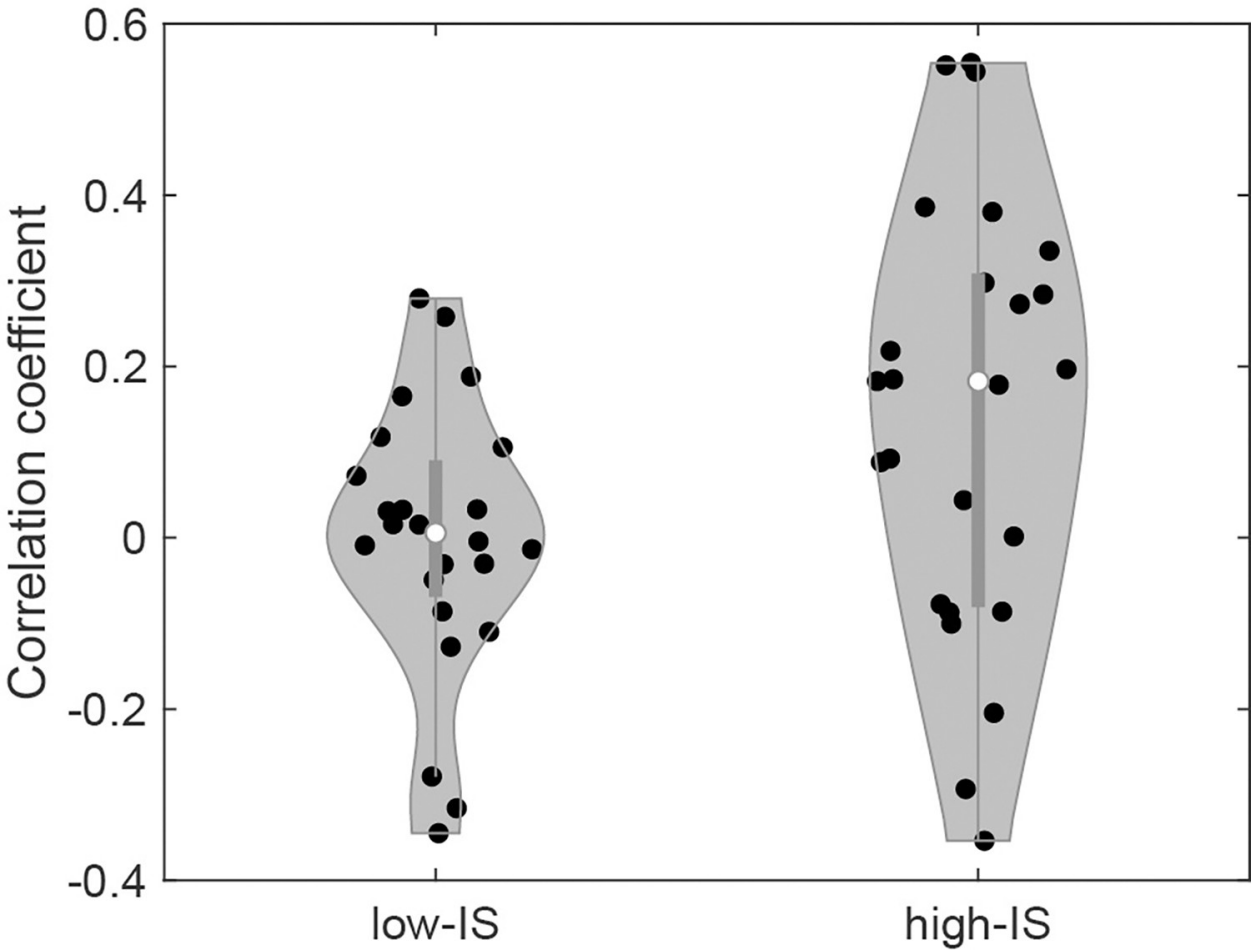
Spearman’s correlation coefficient between valence level and pulse rate. The solid dot indicates each participant and the open dot indicates the median for each group. IS, interoceptive sensitivity.

To further examine whether pulse rate and valence level were linearly correlated, we calculated Spearman’s correlation coefficient between each individual’s valence rating and mean pulse rate. The median of Spearman’s correlation coefficient between the valence rating and the mean pulse rate was 0.17 in the high-IS group and -0.03 in the low-IS group. When the correlation coefficients between the two IS groups were compared by the Kolmogorov–Smirnov test, the correlation coefficient was significantly greater in the high-IS group than in the low-IS group (*p* = 0.013, Fig 3).

We also performed the same analyses based on grouping according to participant IA estimated with the heartbeat counting task (IA factor: *F* (1, 47) = 1.85, *p* = 0.18; valence level factor: *F* (1, 47) = 5.74, *p* = 0.021; IA × valence interaction: *F* (1, 47) = 0.88, *p* = 0.35) and the variance parameter (*σ*) estimated with the heartbeat discrimination task (IS factor: *F* (1, 47) = 0.86, *p* = 0.36; valence level factor: *F* (1, 47) = 5.66, *p* = 0.022; IS × valence interaction: *F* (1, 47) = 2.13, *p* = 0.15); however, none of them showed significant interactions between factors or significant effects in the IS group. Therefore, in the subsequent analyses, we used the heartbeat discrimination task amplitude parameter (*A*) as an index of individual differences in interoceptive sensitivity.

**Fig 4:**
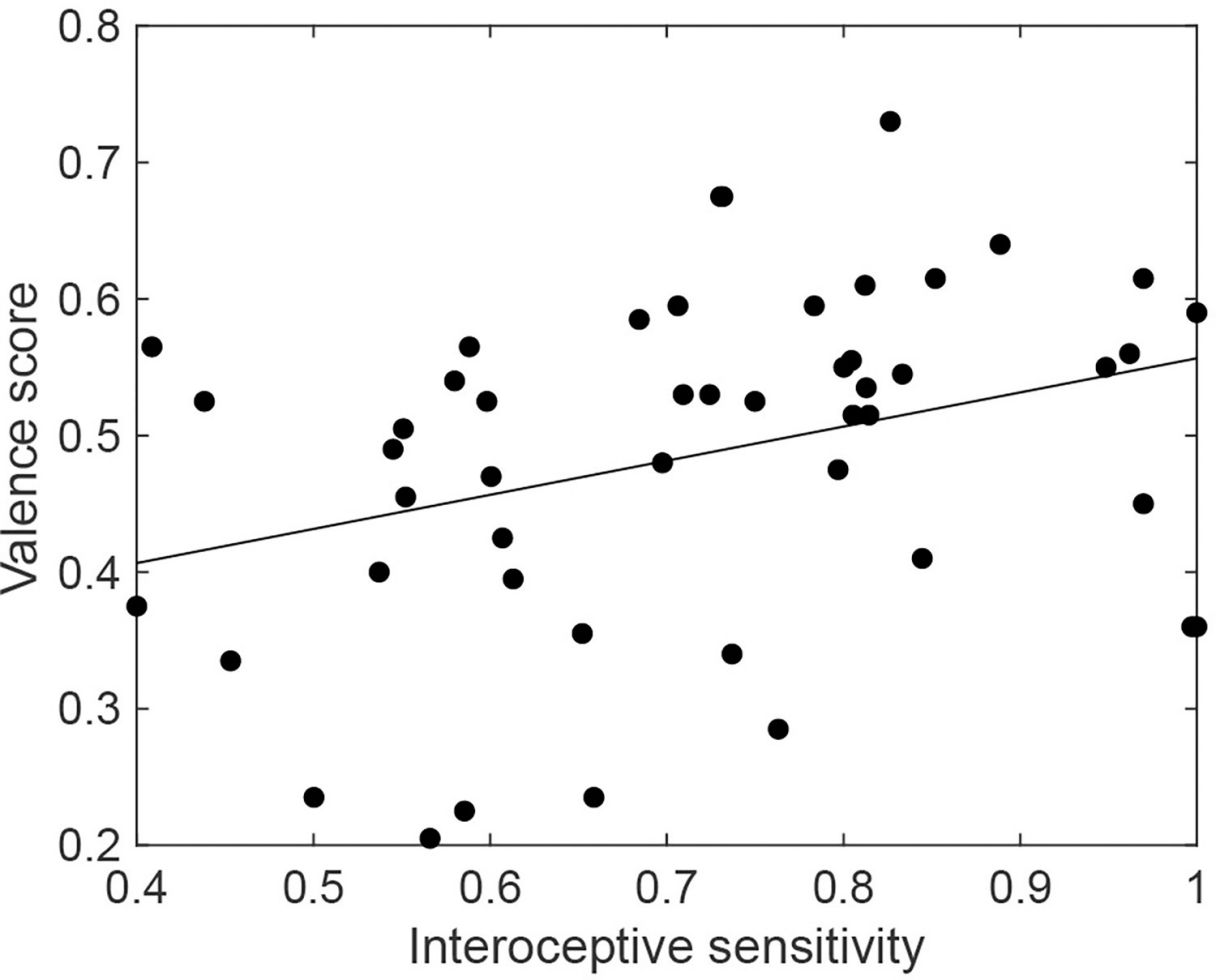
Relationship between the average valence score for all music pieces and interoceptive sensitivity. The relationship between the average valence score for all music pieces and interoceptive sensitivity was defined by the amplitude parameter of the heartbeat discrimination task. Each point indicates each participant’s data. The solid line shows a result of linear regression.

Furthermore, to examine whether the valence score was related to individual differences in interoceptive sensitivity, we conducted a correlation analysis between valence score and interoceptive sensitivity. This analysis revealed a significant positive correlation between interoceptive sensitivity and valence score (*r* = 0.35, *t* (47) = 2.59, *p* = 0.016, Fig 4). In contrast, no significant correlation was found between interoceptive sensitivity and the standard deviation (SD) or the variance of the valence score (SD: *r* = 0.11, *t* (47) = 0.74, *p* = 0.46, variance: *r* = 0.19, *t* (47) = 1.31, *p* = 0.20).

To verify whether the pulse rate changed due to interoceptive sensitivity regardless of valence level, we examined the correlation between pulse rate and interoceptive sensitivity. However, no significant correlation was found between interoceptive sensitivity and the average, SD, or variance of the pulse rate (average: *r* = 0.09, *t* (47) = 0.65, *p* = 0.52; SD: *r* = 0.08, *t* (47) = 0.57, *p* = 0.57; variance: *r* = 0.04, *t* (47) = 0.31, *p* = 0.76).

### Brain activity and interoceptive sensitivity

**Fig 5:**
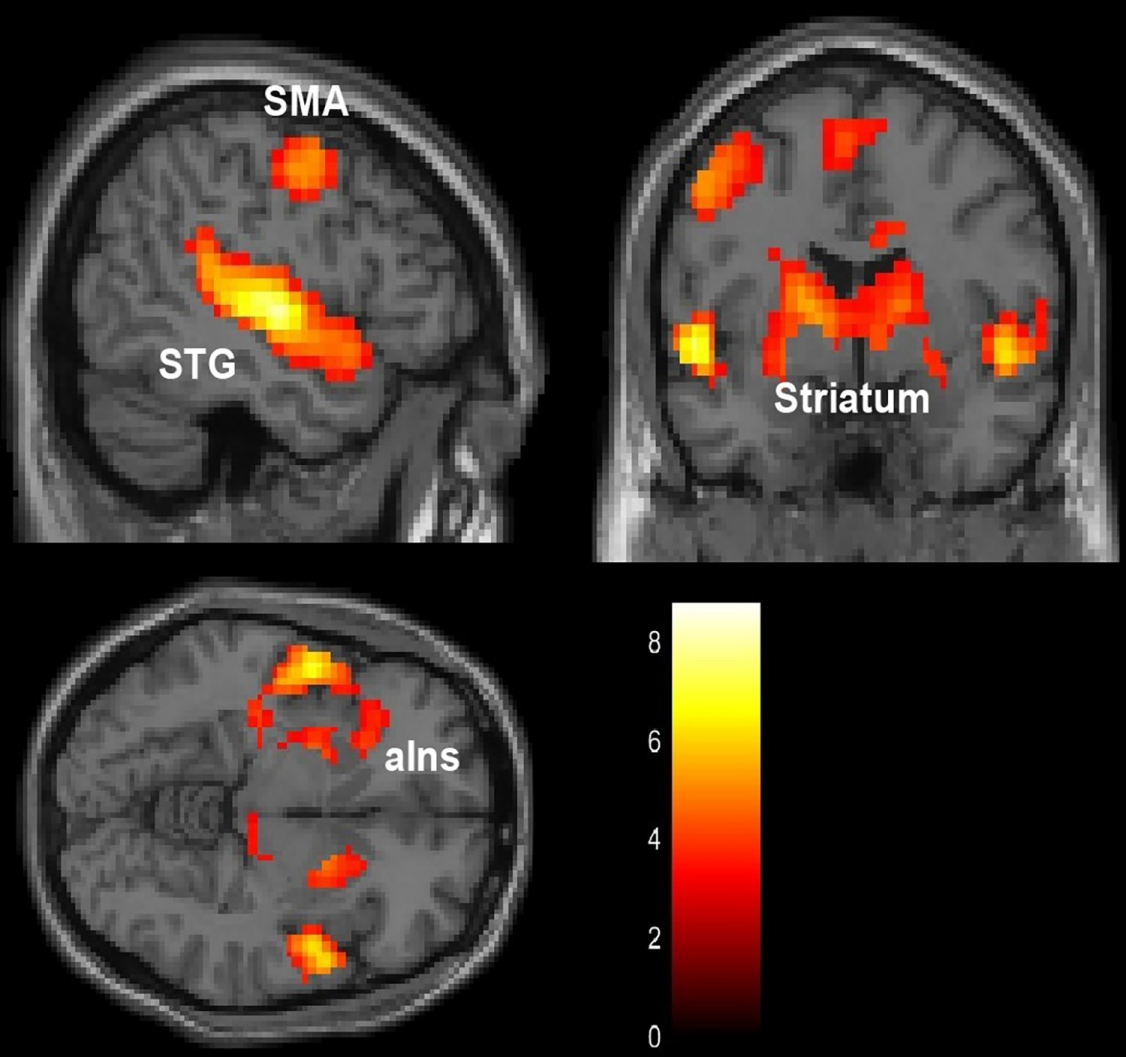
Activations associated with valence level observed by the parametric modulation analysis. Uncorrected *p* < 0.001 at voxel-level, FWE-corrected *p* < 0.05 at cluster level. SMA, supplementary motor area; STG, Superior temporal gyrus; aIns, anterior Insula; FWE, family-wise error.

First, we performed a parametric modulation analysis to identify brain regions whose neural activity was modulated by participant music valence scores. We found significant associations between the valence score and activity in the bilateral auditory cortices (superior temporal gyrus), striatum (caudate nucleus and putamen), primary motor area, left supplementary motor area, right middle cingulate cortex, and left anterior insula (Fig 5, Table 2).

**Table 2:** Anatomical locations and coordinates of brain regions showing significant activations in the high-valence trials.

		Left hemisphere				Right hemisphere			
Cluster	Region	t-score	x	y	z	t-score	x	y	z
**Auditory cortex**	Superior temporal gyrus	8.03	-48	-13	2	8.78	51	-13	2
**Primary motor cortex**	Precentral gyrus	5.14	-51	-1	50	5.71	51	-1	46
**Supplementary motor cortex**	Frontal superior gyrus	6.04	-9	17	46				
**Insula cortex**	Anterior insula cortex	3.76	-33	26	-6				
**Cingulate cortex**	Middle cingulate cortex					3.27	7	0	31
**Striatum**	Caudate	5.53	-15	-4	10	5.21	18	23	10
	Putamen	5.22	-18	8	2	5.18	15	8	-2

The areas with activity significantly positively correlated with affective level. The coordinates are derived from the standard brain of the Montreal Neurological Institute (MNI). The significance level is p < 0.001 without correction at voxel-level, and p < 0.05 with family-wise error (FWE) correction at the cluster level.

Based on the results of whole-brain analysis, which showed significant activity primarily in the left insula when the participants were exposed to music stimuli, we decided to refine our focus in the ROI analysis. The decision to focus on the left insula was informed by our initial observations of lateralized activation patterns and was consistent with the established role of the insula in interoceptive information processing and emotional responses to auditory stimuli (as detailed in the Introduction).

**Fig 6:**
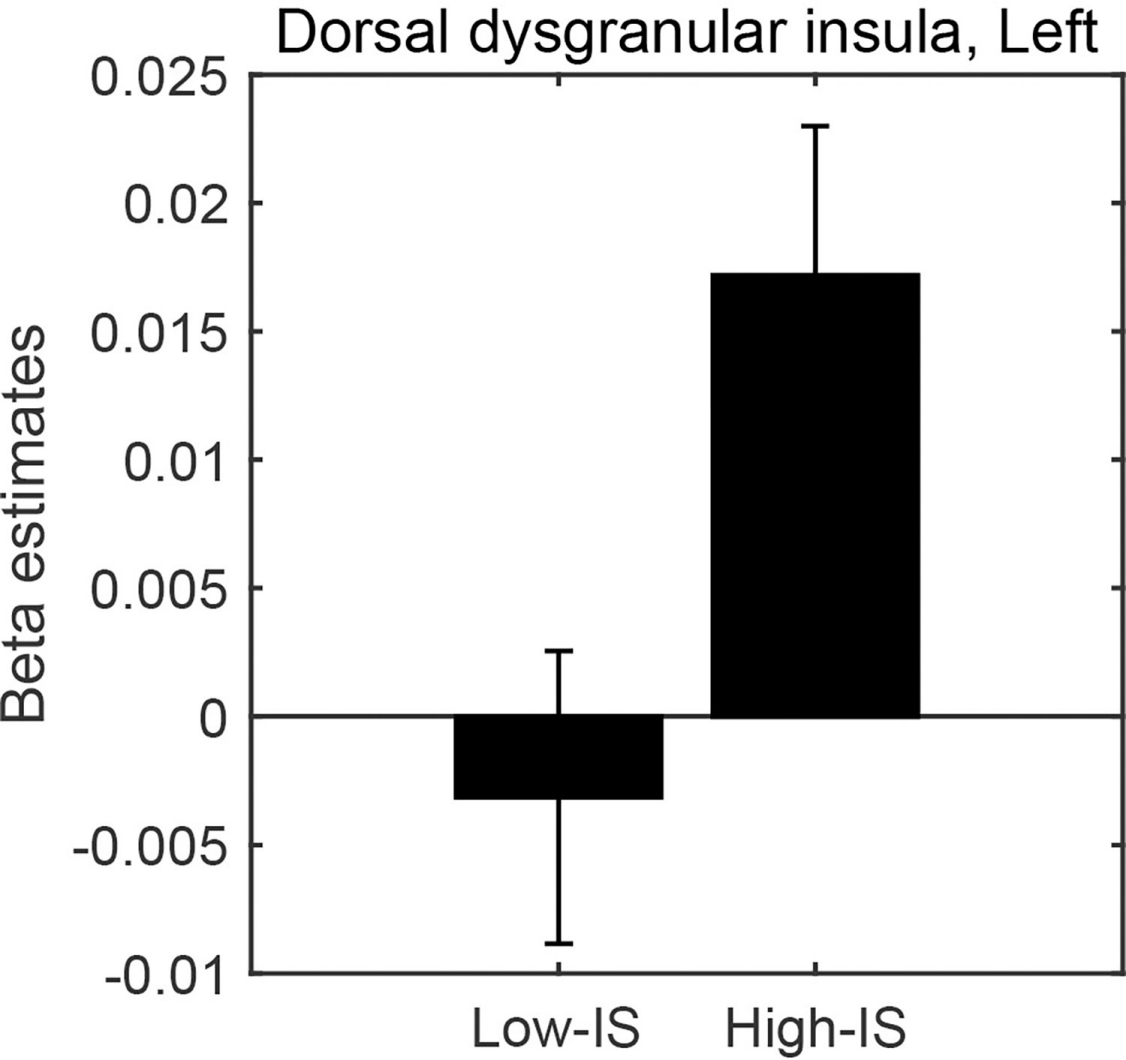
Mean beta values of parametric modulation with valence level in the insula subregion. Each panel represents the insula subregions showing significant differences between the high- and low-IS groups in the ROI (region of interest) analysis. The error bars represent ±1 standard error.

Therefore, we divided the participants into two groups, high-IS and low-IS groups, according to the heartbeat discrimination task parameter (*A*), and compared brain activities between high-IS and low-IS groups for each insula subregion by performing a two-sample *t*-test. This analysis revealed significant differences between the brain activities related to the valence level in the high IS group compared to the low IS group in the left dorsal dysgranular insula (Fig 6, Table 3), suggesting that the correlation between valence level and brain activity in the left mid insula was significantly stronger in the high IS group than in the low IS group.

**Table 3:** Anatomical coordinates of left insula subregions and t-value of ROI analysis.

	t	sig.	x	y	z
Hypergranular insula	0.92		-36	-20	10
Ventral agranular insula	1.20		-32	14	-13
Dorsal agranular insula	1.71		-34	18	1
Ventral dysgranular and granular insula	1.80		-38	-4	-9
Dorsal granular insula	1.22		-38	-8	8
Dorsal dysgranular insula	2.19	*	-38	5	5

The regions of interest were defined using the Brainnetome Atlas. The t-value indicates the result of a one-sided t-test on whether the activity associated with affective level is larger in the high-IS group than in the low-IS group. The asterisk in the table shows p < 0.05 at the significance level corrected with false discovery rate correction for multiple comparisons. ROI, region of interest.

## Discussion

The present study aimed to investigate two open questions related to the neural mechanisms underlying the relationship between interoceptive sensitivity and emotional responses to music stimuli; namely, whether individual differences in interoceptive sensitivity influence the relationship between subjective emotional intensity and physiological signals in response to music stimuli and the involvement of specific insula subregions in emotion processing.

Using our new index of interoceptive sensitivity (amplitude parameter *A*) derived from the heartbeat discrimination task, we were able to demonstrate that participants with high interoceptive sensitivity (high-IS group) displayed an increase in pulse rate when listening to high-valence music pieces (high-valence trials). This finding supports the first hypothesis that the physiological response during an emotional experience correlates with individual differences in interoceptive sensitivity, which was estimated using the heartbeat discrimination task.

fMRI analysis revealed that the valence level was significantly associated with increased activity in the left anterior insula. Furthermore, ROI analysis of the insula subregions revealed that the high-IS individuals showed significantly stronger activity in the dorsal dysgranular insula. These results indicate that posterior insula activity during an emotional experience correlates with the individual difference in the interoceptive sensitivity estimated by the heartbeat discrimination task, supporting the second hypothesis.

### Method for measuring interoceptive sensitivity

To address the complexity of measuring interoceptive sensitivity, our study took a novel approach to the heartbeat discrimination task. This was informed by literature suggesting that participants often perceive a shift from a 0-ms condition as synchronous [38,40], leading us to compute an independent threshold deviating from the subjective equivalence point shift. In addition, the inherent variability observed in the data led us to use a parametric approximation—specifically, a Gaussian function—to estimate this threshold.

This study also included the heartbeat counting task. Previous studies of emotional and physiological responses have used heartbeat counting tasks to estimate interoceptive sensitivity. However, this method has been criticized because participant knowledge of their heart rate affects their scoring [34–36]. The heartbeat discrimination task provides a more accurate measure of participant sensitivity to heart rate because it minimizes the influence of participant knowledge and scoring strategies [38,40]. When comparing the two tasks, we found a positive correlation (*r* = 0.34) between IA and amplitude alone for the heartbeat discrimination task.

In previous studies, the correlation between heartbeat counting and heartbeat discrimination tasks has been controversial; some researchers have reported positive correlations [37,44–46], while others have reported no correlation [38,47,48]. An integrative review of these studies suggests a weak correlation of 0.20 [49], which is close to that of the present study. Contrary to previous studies, we developed two new heartbeat discrimination indices (*A* and *σ*).

The amplitude index A is proposed to reflect the signal-to-noise ratio of heartbeat perception, potentially indicating individual sensitivity to heartbeat intensity. The variance index σ is intended to represent the temporal resolution of heartbeat perception, possibly illustrating the capacity for temporal integration with exteroceptive senses, such as auditory perception. The significant correlation observed between the IA index (estimated using the heartbeat counting task) and the amplitude index A (estimated using the heartbeat discrimination task) suggests that the heartbeat counting task is effective in detecting individual differences in heartbeat perception ability.

Regarding the relationship between the music listening task and the heartbeat tasks, a significant difference in pulse rate was observed only when the participants were grouped according to the amplitude parameter derived from the heartbeat discrimination task. One reason for this is that the amplitude of the heartbeat discrimination task may have been more strongly related to participant music valence ratings.

The heartbeat counting task does not require any exteroceptive sensation, since the participant simply counts heartbeats. In contrast, the heartbeat discrimination task requires participants to compare their heartbeats to the simultaneously presented tones. Thus, the heartbeat discrimination task requires integrated processing of interoception and exteroception. Notably, the generation of emotion requires the integration of interoception and exteroception [50,51]; thus, the ability to integrate interoception and exteroception may be strongly associated with the physiological response to music.

### Individual differences in interoceptive sensitivity and physiological response

A comparison of the pulse rates when listening to music among the high-valence, middle-valence, and low-valence trials showed that the higher the valence rating, the higher the pulse rate. This is consistent with the typical tendency observed when listening to music or feeling emotions induced by music [52]. Furthermore, in the present study, we found a relationship between changes in pulse rate and individual differences in interoceptive sensitivity: the pulse rate was higher when listening to the high-valence music piece only in the high-IS group.

This observation aligns with previous research demonstrating a correlation between interoceptive awareness and the intensity of physiological responses, triggered by visual stimuli affecting emotional perception [10–12,14]. However, no significant change in pulse rate was observed when we analyzed trials grouped by the type of music pieces (tonal, atonal, and dissonance). This suggests that subjective evaluation is more related to the pulse rate than the type of music. In addition, there was a weak positive correlation between interoceptive sensitivity and the average valence score, but no significant correlation between interoceptive sensitivity and valence score variance, average pulse rate, or pulse rate variance. This indicates that the participants with low and high interoceptive sensitivity showed similar changes in valence score and pulse rate.

There are two possible interpretations for the finding of a relationship between valence score and pulse rate only in the high-IS group. First, the pulse rate of individuals with high IS fluctuates greatly depending on their emotions. Second, high-IS individuals refer to their pulse rate when evaluating emotions. If the first hypothesis was true, the participants in the low-IS group would show less substantial pulse rate changes among the trials.

However, we observed no difference in the variance of pulse rate changes between the high- and low-IS groups, which indicates that the second interpretation is appropriate. Based on our findings, we can speculate that individuals with high interoceptive sensitivity might use their physical condition to assess their emotional level when listening to music, while those with low interoceptive sensitivity might use their knowledge of moving and non-moving music or experience of emotional music.

Dunn and Galton [53] showed that participants with high interoceptive accuracy had a greater effect of anticipatory EDA on risk-aversion behavior. However, there was no difference in the magnitude of EDA depending on interoceptive accuracy. In other words, when making a risky selection, EDA occurs in the same way regardless of the interoceptive accuracy, but an individual with high interoceptive accuracy can effectively utilize it for selection behavior.

Consistent with this result, in the present study, individuals in the high-IS group showed a large pulse rate change during the high-valence trials, although the magnitude of the pulse rate change did not differ according to interoceptive sensitivity. Therefore, the magnitude of the physiological response does not change depending on individual differences in interoception. The individual differences in interoception might not be associated with differences in the magnitude of the physiological response, rather with the difference in the use of the physiological response for evaluating emotions.

### Brain activity and interoceptive sensitivity

In our whole-brain fMRI analysis, we observed associations between music valence scores and activity in the bilateral auditory cortices (superior temporal gyrus), striatum (caudate nucleus and putamen), primary motor area, left supplementary motor area, right middle cingulate cortex, and left anterior insula. Notably, the role of the anterior insula is prominent for interoceptive sensitivity [16,21,51], suggesting its importance in emotional processing.

The bilateral auditory cortices—particularly the superior temporal gyrus—play a central role in the perception of music, involving the processing and understanding of sound [54]. This is consistent with our findings, as higher music valence scores were associated with increased activity in these areas, suggesting their involvement in the emotional aspects of music perception. In addition, the striatum, which includes the caudate nucleus and putamen, has been implicated in reward processing and emotional responses that reflect the pleasure and satisfaction derived from music [55,56]. This suggests that the activity of the striatum could be indicative of the pleasure that participants experience while listening to music, consistent with the emotional valence of the pieces.

The primary motor area and left supplementary motor area are known to be involved in motor responses and musical rhythm synchronization [57]. This may explain the activation of these areas in response to high-valence music, potentially reflecting the physical embodiment of musical rhythm and its emotional impact.

Additionally, the right middle cingulate cortex, which is involved in attention and emotional regulation, could influence emotional processes during music listening [58], perhaps by modulating the intensity and focus of emotional responses to music. Thus, the current findings support the notion that the physiological and emotional responses elicited by music involve the functions of brain regions that represent interoceptive, somatic, and emotional states. However, further research is necessary to elucidate the specific roles of each of these regions and their functional connections, particularly in terms of how they represent general emotional intensity and subjective emotional experiences in response to music.

In our subsequent ROI analysis, we focused specifically on the left anterior insula, given its significant activity in the whole-brain analysis and its prominent role in proprioceptive sensation and emotional processing. The ROI analysis on the insula subregions showed significantly higher activity in the left dorsal dysgranular insula in the high-IS compared to the low-IS groups. The anterior insula has been associated with the integration of perceptions, emotions, thoughts, and plans, contributing to a subjective representation of “our world.” The posterior insula, on the other hand, is more closely associated with bodily sensorimotor functions, including those related to internal organs [59].

The anterior insula is connected to the orbitofrontal, cingulate, and limbic cortices, while the posterior insula is connected to the parietal area, such as the somatosensory and motor cortices [60–63], which correspond to their respective functions. Barrett and Simmons [51] proposed a model of interoceptive processing focusing on the composition of the insula subregion and argued that the granular cortex receives the interoceptive sensory input, while the agranular cortex provides interoceptive predictions. Since the posterior insula, which showed a significant difference in activity between the high- and low-IS groups, receives sensory input, it is presumed that participants with high interoceptive sensitivity receive more interoceptive sensory input during the emotional experience. From the results of the physiological responses, we can speculate that participants with high interoceptive sensitivity received strong heartbeat sensory input and used it for emotional evaluation, and the strong sensory input may have been reflected in the activity of the posterior insula.

The mid insula connects the anterior and posterior regions, and it has been reported that the brain waves generated by pain are transmitted from the posterior insula to the anterior insula via the mid insula [64]. Kuehn and Mueller [30] also showed that interoceptive sensitivity is related to the strength of the connection between the posterior insula and the mid/anterior insula. The significant correlations found in the present study between dorsal dysgranular insula cortical activity and emotional experience in participants with high interoceptive sensitivity are consistent with the findings of this study. Although these results suggest that the mid insula may play a role in integrating physiological signals and music stimuli to produce emotional experiences, definitive conclusions about the involved neural pathway and its exact function require further investigation.

### Limitations

Our study of interoceptive sensitivity, heart rate, and insula activity in response to music has several limitations that warrant attention. A main limitation comes from our methodological decision to introduce novel indices, parameters A and σ, into our heartbeat discrimination task. While these indices provide a unique perspective for assessing interoceptive sensitivity, their use may limit the ability to directly compare our findings with those of other studies. This choice highlights the need for further validation and comparison with well-established methods in future research.

Another important limitation concerns the measurement of emotional responses. Our study used the Japanese concept of “Kando,” which focuses on the intensity of positive emotional responses to music. However, this approach did not allow us to distinguish between positive and negative emotions, thus limiting our ability to analyze and interpret the qualitative aspects of the emotional responses elicited by different types of music.

Furthermore, the generalizability of our findings is limited by the specific demographics of the healthy volunteers who participated in the study. This participant pool may not be fully representative of the broader population, suggesting the need for future studies to include a more diverse range of participants to explore the generalizability of these findings.

These limitations are crucial for contextualizing our findings and should guide future research efforts aimed at deepening our understanding of the intricate relationship between interoceptive sensitivity, emotional response, and musical experience.

### Conclusions

In the present study, we investigated the relationship between individual differences in interoceptive sensitivity, ratings of emotional valence level, and pulse rate when listening to music. We found an increased pulse rate in high-valence music trials for the high-IS group only, which was defined by the amplitude parameter of the heartbeat discrimination task. This suggests that the internal physiological state of individuals with high interoceptive sensitivity may influence the assessment of their emotional experience when listening to music.

We also found no significant relationship between pulse rate and interoceptive sensitivity assessed with the heartbeat counting task, suggesting higher reliability of the heartbeat discrimination to assess interoceptive sensitivity and its relationship with the physiological response of emotional experience. Furthermore, our ROI analysis showed that dorsal dysgranular insula activity was significantly correlated with emotional experiences in individuals with high IS than in those with low IS, while no such association was observed with the anterior insula. This suggests that the mid insula may be the locus where primary interoception representations and physiological signals interact with music-induced stimuli to generate an emotional experience. These results extend the knowledge of interoceptive processing and elucidate the role of interoception in emotions.

## Supporting information

S1 File (M)

S2 File (M)
